# Arsenic Speciation and Metallomics Profiling of Human Toenails as a Biomarker to Assess Prostate Cancer Cases: Atlantic PATH Cohort Study

**DOI:** 10.3389/fpubh.2022.818069

**Published:** 2022-07-07

**Authors:** Erin Keltie, Kalli M. Hood, Yunsong Cui, Ellen Sweeney, Gabriela Ilie, Anil Adisesh, Trevor Dummer, Veni Bharti, Jong Sung Kim

**Affiliations:** ^1^Department of Community Health and Epidemiology, Faculty of Medicine, Dalhousie University, Halifax, NS, Canada; ^2^Health and Environments Research Centre (HERC) Laboratory, Faculty of Medicine, Dalhousie University, Halifax, NS, Canada; ^3^Atlantic Partnership for Tomorrow's Health (PATH), Dalhousie University, Halifax, NS, Canada; ^4^Division of Occupational Medicine, Department of Medicine, University of Toronto, Toronto, ON, Canada; ^5^Department of Medicine, Dalhousie Medicine New Brunswick, Saint John, NB, Canada; ^6^School of Population and Public Health, University of British Columbia, Vancouver, BC, Canada

**Keywords:** arsenic exposure, metallome, speciation, toenail biomarker, prostate cancer, cohort study

## Abstract

Chronic exposure to inorganic arsenic and trace metals has been linked to prostate cancer, and altered arsenic methylation capacity may have an important role in arsenic carcinogenesis. Biomarkers may be able to elucidate this role. Our objectives were to characterize profiles of arsenic species and metallome in toenails and urine samples, compare profiles between prostate cancer cases and controls, and determine the discriminant ability of toenail and urine biomarkers. Toenail samples (*n* = 576), urine samples (*n* = 152), and questionnaire data were sourced from the Atlantic Partnership for Tomorrow's Health (PATH) cohort study. Healthy controls were matched to prostate cancer cases (3:1 ratio) on sex, age, smoking status, and the province of residence. Metallome profiles and proportions of arsenic species were measured in toenail and urine samples. Analysis of covariance (ANCOVA) was used to compare the mean percent monomethylarsonic acid (%MMA), dimethylarsonic acid (%DMA), inorganic arsenic (%iAs), primary methylation index (PMI, MMA/iAs), and secondary methylation index (SMI, DMA/MMA). Multivariate analysis of covariance (MANCOVA) was used to compare selected metal concentrations. Mean %MMA was significantly lower and SMI was significantly higher in toenails from prostate cancer cases compared to controls in unadjusted and adjusted models. Proportions of arsenic species were correlated with total arsenic in toenails. Arsenic speciation in urine was not different between cases and controls, nor were metallome profiles in toenails and urine. Our results indicate that toenails are a viable biomarker for altered arsenic speciation in prostate cancer cases and may have greater utility than urine in this context.

## Introduction

Elemental arsenic (As) and inorganic arsenic (iAs) compounds have been classified as Group I carcinogens by the International Agency for Research on Cancer (IARC). The IARC found that there is sufficient evidence (IARC group 1) in humans that exposure to iAs has a causal relationship with cancer of the lung, urinary bladder, and skin, and is associated with prostate cancer among others (1). The main source of iAs exposure for the general population is through contaminated drinking water, where it primarily exists in inorganic forms (2, 3), and consumption of food sources with an affinity for iAs uptakes such as rice, other cereals, shellfish, and seaweed (4). There may be a dose-dependent relationship between chronic low-level exposure to iAs in drinking water and prostate cancer (5). Due to its association with cancer and other adverse health events (6), governing agencies have recommended As not exceed 10 μg/L in drinking water (7–9). In Atlantic Canada (New Brunswick, Newfoundland and Labrador, Nova Scotia, and Prince Edward Island), residents are particularly vulnerable to iAs exposure given the elevated iAs concentrations found in the bedrock. In addition, about half of the provinces' residents rely on private wells for domestic water supply (10, 11). In Canada, As concentration in private well water is not regulated by the government, and the responsibility of testing and remediation falls to the homeowner. However, compliance is low and previous studies have found that many of the private wells in this area exceed the Health Canada Maximum Acceptable Concentration (MAC) limit of 10 μg/L (7, 12). In addition to elevated iAs, Atlantic Canada has the highest age-standardized incidence rates of cancer in the country (13) and is projected to have the highest age-standardized cancer mortality rates (14). Considered together, these conditions make Atlantic Canada an ideal location to investigate associations between chronic iAs exposure and prostate cancer.

Metals can be essential or toxic (15) depending on their dose and physiological effects. For example, zinc (Zn) and magnesium (Mg) are necessary for biological functions (16), whereas exposure to cadmium (Cd) by inhalation is carcinogenic (1). Research has recently shifted away from individual metal assessment toward a more comprehensive metallomics approach, which acknowledges the complex interplay of metal functions within the body (17). Dose-response curves indicate that there may be an optimal level to maintain physiological benefits whilst avoiding effects from deficiency or excess, even for essential elements (16).

With respect to iAs, chronic exposure may lead to carcinogenesis in multiple ways. Proposed mechanisms of effect include oxidative DNA damage (18, 19), inhibited DNA repair (20, 21), and altered gene expression (22, 23). Impaired DNA repair and chromosomal instability can induce genotoxicity, resulting in mutations of tumor suppressor genes (24) and carcinogenesis (25). Together, these conditions are favorable for the uncontrolled proliferation of cancerous cells.

Although the mechanisms of iAs metabolism are not yet fully understood, they are also thought to play an important role in carcinogenesis. The As metabolism process results in iAs being converted to monomethylarsonic acid (MMA^V^) and subsequently dimethylarsenic acid (DMA^V^) *via* successive methylation (26–29). The process produces intermediate metabolites (iAs^III^, MMA^III^, and DMA^III^) that may be more reactive and toxic (29). This metabolism pathway results in a mixture of As species being excreted in urine or deposited in keratin-rich materials, such as hair and nails (30). The ability to methylate iAs or the differential presentation of As metabolites may be key in understanding cancer risk related to iAs exposure as evidenced by observed inter-individual differences in the capacity to methylate iAs. Some of these differences may be partially attributed to the total amount of As exposure, which has been positively associated with %MMA in urine (31, 32). Furthermore, chronic iAs exposure from drinking water has been associated with decreased capacity to methylate iAs to DMA and increased risk of As-related disease (33–35). Reduced iAs methylation ability has also been linked with an increased risk of breast cancer (36) and bladder cancer (37, 38). The remaining differences observed in methylation capacity at the population level may be explained by genetic differences (32), whereas individual variation may be related to age and sex. Specifically, methylation capacity may decrease with age, and women methylate As more efficiently than men (39, 40).

The current literature is lacking the comprehensive approach required to elucidate associations between exposure to iAs and other trace metals, iAs methylation capacity, and cancer. The use of metallomics and As speciation profile analyses consider the complex interactions of metals and the role of iAs methylation, respectively, and improves upon existing research beyond a univariate assessment. Moreover, there are clear differences across populations – as a result of different genetic composition, lifestyle factors, exposure levels, or some combination – highlighting the need for analyses of different population groups and the consideration of external factors.

Urine is a well-established biomarker and represents an accurate representation of acute As exposure, with approximately 75% of absorbed As excreted through urine (41). Similar to urine, metal concentration in nails has also been shown to be a proxy for exposure (42–45). Due to their slow growth rate, toenails reflect a longer period of exposure than urine, and As concentrations remain more stable over time (46). Since As-induced diseases often arise from chronic long-term exposure, toenails may be an appropriate biospecimen for use in investigating the association between chronic iAs exposure and prostate cancer. To date, most epidemiologic studies investigating risks associated with exposure to iAs, other semimetals, and metals have measured the concentrations of metals and As speciation in urine. Among studies that have used nails as a biomarker and focused on total metal concentrations, the evidence is conflicting (47). Furthermore, studies that have focused on As speciation in nails did not investigate the potential association with cancer development (48–50).

The body burden as represented by an As speciation profile or “fingerprint” in individuals affected by As-related cancers is not well understood and data are lacking for toenails as biospecimens. Prostate cancer was chosen as the focus of this study due to the availability of biospecimens, with the goal of expanding to other cancer types in the future after confirmation of feasibility. The overall aim of this study was to compare the profiles of As species and metallome between prostate cancer cases and healthy controls from the Atlantic PATH cohort study and assess the utility of toenail and urine samples for biomonitoring. This was achieved through the following objectives: 1) Measuring total concentrations of As and other metals and the levels of As species in toenails and urine samples among prostate cancer cases and controls (toxicological laboratory analysis), and 2) Assessing the biomarkers' abilities to capture differences in profiles between prostate cancer cases and controls using matched toenail and urine samples (statistical analysis).

## Methods

### Study Design and Population

**Figure 1:**
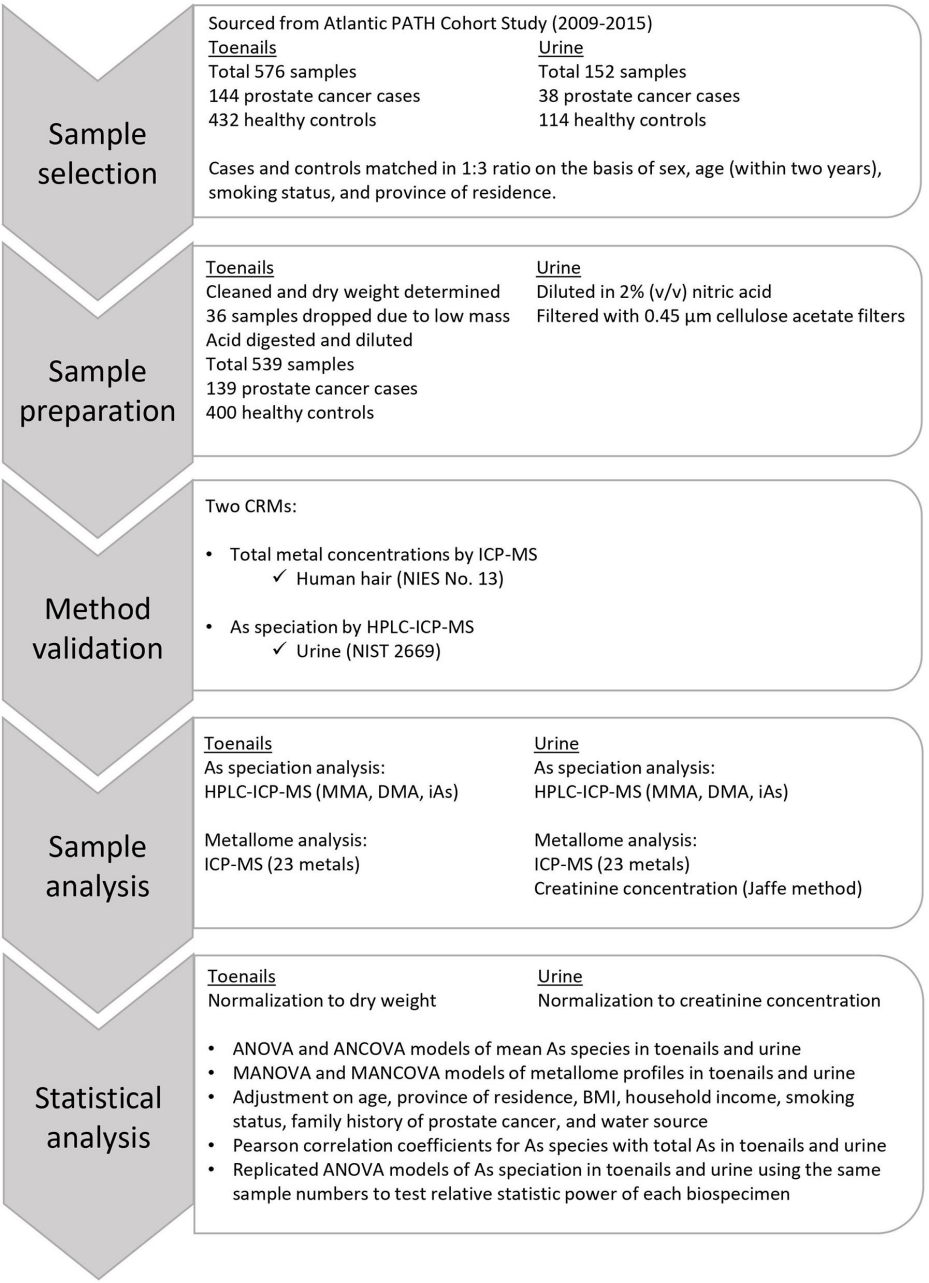
Flowchart of the approach and methods used in this study.

The methods and approach used for this research are depicted in Figure 1. The data used in this study were sourced from the Atlantic PATH cohort, which is a part of the larger Canadian Partnership for Tomorrow's Health (CanPath, formerly the Canadian Partnership for Tomorrow Project). CanPath is the largest prospective cohort in Canada with more than 330,000 participants across the country, examining the contributions of genetic, environmental, lifestyle, and behavioral factors to the development of cancer and chronic disease (51). The Atlantic PATH cohort has recruited over 35,000 participants from Atlantic Canada to provide questionnaire data, physical measurements, and biospecimens including blood, urine, saliva, and toenail samples. The Atlantic PATH cohort study is described in greater detail in Sweeney et al. (52). All available prostate cancer case toenail and urine samples from the Atlantic PATH baseline collection (2009–2015) were included in this study, and control samples were randomly selected to match the case samples by sex, and age within 2 years, province of residence, and smoking status. The Atlantic PATH participants are representative of the Atlantic Canadian population which is primarily White (52).

### Analytical Methods

Analytical methods were established and validated prior to the present analyses (53), are provided in full detail in Supplementary Materials, and have been summarized here.

#### Sample Preparation

Toenail clipping samples were weighed to determine a wet weight of approximately 50 mg. After weighing, toenail clipping samples were transferred to 10 mL quartz digestion vessels and cleaned by sonicating in acetone for 5 min, rinsing with acetone, then sonicating in water for 5 min. Samples were subsequently rinsed with water three times before being dried at 105°C overnight. Once dry, samples were reweighed to determine dry mass. Toenail clipping samples were dissolved using a Discover SP-D microwave digester (CEM Corporation, NC, USA) in 1 mL of a solution comprised of 10% v/v nitric acid, 40% v/v hydrogen peroxide, and 50% v/v deionized water. After digestion, samples were diluted to 10 mL with deionized water for a final nitric acid concentration of 1% (v/v), then transferred to 15 mL polypropylene tubes. For As species determination, a 995 μL aliquot of each sample was transferred to a 1.8 mL polypropylene vial, and 5 μL of 20 μg/L arsenobetaines (AsB, Sigma Aldrich, MO, USA) in 1% v/v nitric acid was added as an internal standard. Samples were vortexed immediately before analysis to ensure homogeneity.

Urine samples were first vortexed, then a 5 mL aliquot was diluted with 1% (v/v) nitric acid to a volume of 2.5 mL. Urine samples were filtered through 0.45 μm cellulose acetate filter (Sarstedt, NC, USA) before being added to a 15 mL polypropylene tube and diluted with 1% (v/v) nitric acid to a final volume of 5.5 mL. For As species analysis, a 1 mL aliquot of each diluted sample was transferred to a 1.8 mL polypropylene vial. Samples were vortexed immediately before analysis to ensure homogeneity.

#### Creatinine Measurement

Creatinine concentration in urine was measured using Jaffe's method (see Supplementary Materials). An eight-point calibration curve was prepared using a serial dilution of creatinine standard (Sigma-Aldrich, ON, Canada) in deionized water between 0.31 mg/dL and 20 mg/dL. Urine samples were diluted 40 times. A 50 μL volume of standards, urine samples, and method blanks were added to 100 μL of alkaline picric acid solution (Sigma-Aldrich, ON, Canada) in 96-well plates (VWR, ON, Canada), then incubated at room temperature for 30 min. Absorbance was measured at 490 nm using a microplate reader (Synergy H1, BioTek, VT, USA).

#### Metallome Analysis

The concentrations of 23 metals (Li, V, Cr, Mn, Fe, Co, Ni, Cu, Zn, Ga, As, Se, Rb, Sr, Ag, Cd, Sb, Ba, Hg, Tl, Pb, Th, and U) in toenail clipping and urine samples were measured using an inductively coupled plasma-mass spectrometer (ICP-MS, iCAP Q, Thermo Scientific, MA, USA). The sample introduction system consisted of an ESI SC-4 DX autosampler and FAST valve (Elemental Scientific, NE, USA) to facilitate the online addition of a 50 μg/L Sc internal standard (AccuStandard, CT USA) in 1% (v/v) nitric acid. Measurements were performed in kinetic energy discrimination (KED) mode using high purity He (> 99.999%) as the collision gas. Qtegra Intelligent Scientific Data Solution software (version 2.7, Thermo Scientific, MA, USA) was used to collect and process data from ICP-MS analysis.

#### Arsenic Speciation Analysis

Arsenic speciation analysis was completed using high-performance liquid chromatography (HPLC, SpectraSYSTEM, Thermo Scientific, MA, USA) hyphenated with an ICP-MS. The injection of each sample solution and subsequent measurement was coordinated using the Qtegra Intelligent Scientific Data Solution software (version 2.7, Thermo Scientific, MA, USA). An anion exchange column (IonPac AS7, 250 mm x 2 mm, Thermo Scientific, MA USA) and a guard column (IonPac AG7, 50 mm x 2 mm, Thermo Scientific, MA, USA) were used to separate As species in toenail clipping and urine samples. Ammonium carbonate was used as the mobile phase with a gradient solution between 20 mM and 200 mM to achieve good separation for all As species measured. The use of hydrogen peroxide for toenail digestion was assumed to have converted As^3+^ to As^5+^, as no As^3+^ was detected in toenail samples, which was confirmed using certified reference materials (CRMs).

#### Method Validation

A total of two CRMs were employed to validate the results of total metal measurement by ICP-MS and As speciation analysis by HPLC-ICP-MS: NIES No. 13 Human Hair (National Institute for Environmental Studies, Tsukuba, Japan), described by Yoshinaga et al. (54) and NIST 2669 Arsenic Species in Frozen Urine (National Institute of Standards and Technology, ML USA), described by Davis et al. (55). These materials were considered the best analogs for toenails because there were no CRMs for metals nor As speciation in toenails at the time of analysis. Preparation methods are detailed in Supplementary Materials and results are reported in Supplementary Table 1. The method detection limits (MDLs) were calculated following United States Environmental Protection Agency (USEPA) procedures (56). Seven method blanks of 1% v/v nitric acid carried through the entire sample preparation procedure alongside toenail samples were used to calculate the MDL for metals and As species. The blanks did not contain MMA nor DMA, so seven replicates of the lowest level calibration standard (0.02 μg/L in 1% v/v nitric acid) were used to calculate the MDL for MMA and DMA. Details are provided in Supplementary Materials and MDLs are reported in Supplementary Table 2. Results of As speciation analysis of NIST 2669 following digestion with nitric acid and hydrogen peroxide confirmed that As^3+^ species are converted to As^5+^ during digestion.

### Statistical Analysis

Differences in study participant characteristics between prostate cancer cases and controls were compared with mean and standard deviation (*SD*) for continuous variables, and frequency and percentage for categorical variables. Analysis of covariance (ANCOVA) and multivariate ANOVA (MANOVA) were chosen to compare profiles of As species and metallome respectively between cases and controls. ANCOVA models were chosen for As speciation profiles because the variables may be interrelated, to account for the inflated type I error, a Bonferroni adjustment was made to the significance level (α =0.05/((number of comparisons)) =0.01). MANOVA was selected for metallome profiles because the variables are not significantly correlated, and it reduces the likelihood of type I error from multiple tests. Model significance was evaluated using Wilks' lambda criterion, which is presented alongside estimated mean difference with accompanying *p*-values and effect size expressed as partial η^2^ [${\eta^{2}}_{p}$, (57)]. Individual metal variables were selected for MANOVA using Student's *t*-test or the Wilcoxon test defined *a priori*, with model inclusion criteria being either a reported association with prostate cancer or *p* < 0.1.

In both ANCOVA and MANOVA models, mean differences in i) As species and methylation capacity measures and ii) total metallome were compared between prostate cancer cases and controls with crude and adjusted analyses. In the adjusted model, covariates were selected if they were associated with the outcome of prostate cancer diagnosis and included age, province, body mass index (BMI), smoking status, family history of prostate cancer, and water source. We utilized logistic regression models to compute odds ratios (OR) and 95% CIs for the binary outcomes of prostate cancer for per *SD* change of As speciation measures and selected metal concentration in toenail and urine samples. In the multivariable regression analysis, we adjusted for age, province of residence, household income, BMI, smoking status, family history of prostate cancer, and water source. Pearson correlation coefficients were calculated to determine whether total As concentration was linked with As species and methylation capacity measures. These analyses were performed independently in our urine and toenail datasets.

A separate, secondary analysis using an equal number (*n* = 140) of toenail and urine samples was performed to compare the relative statistic power of measuring As speciation in toenails and urine to investigate if the different sample size (*n* = 539 toenails and *n* = 152 urine) causes the significance of As speciation profiles in toenail samples from prostate cancer cases. This analysis replicated the previously described ANCOVA models among a subset of the participants who provided both urine and toenail samples. Data management and analyses were performed with SAS statistical package version 9.4 (SAS Institute, NC, USA). Unless otherwise specified, all tests were two-sided and evaluated with α = 0.05. Questionnaire data were categorized, and missing observations were labeled unknown in the adjusted ANCOVA and MANOVA models.

## Results

Among the 576 participants with toenail clipping samples, three samples had insufficient mass (<0.1 mg) to be quantitatively weighed and 19 samples had insufficient mass (<5 mg) for ICP-MS analysis. Each remaining case sample was randomly matched to at least one corresponding control on the basis of sex, age (within 2 years), smoking status, and the province of residence, 123 cases were matched with 3 controls, for 15 cases, matching was only possible with 2 controls, and 1 case matched with 1 control. After the matching process, 139 prostate cancer cases and 400 healthy controls were included in the statistical analysis of toenail samples. All 38 prostate cancer cases and 114 control samples were included in the analysis of urine samples.

### Demographic Characteristics

Participant age at the time of toenail sample collection ranged from 49 to 71 years for cases and 48 to 70 years for controls. The mean age of participants that provided toenail samples was 61.7 years for cases and 61.2 years for controls. Approximately half (49.5%) of all participants (70 cases, 197 controls) were from New Brunswick, and participants from Newfoundland and Labrador comprised 22.8% of samples (31 cases, 92 controls), 22.4% of samples (31 cases, 90 controls) were from Nova Scotia, and 5.2% of samples (7 cases, 21 controls) were from Prince Edward Island. Half (50.7%) of participants provided information regarding primary water sources, among which 34% reported municipal or bottled water and 16.7% reported well water. Overall, 13.0% of participants had a family history of prostate cancer. Prostate cancer cases had a higher percentage of family history of prostate cancer (25.2%) compared to healthy controls (8.8%). BMI information was missing for 30.1% of samples (29.5% of cases and 30.3% of controls). Among those for whom data were available, mean BMI was 29.1 (*SD* = 4.5) overall, 29.6 (*SD* = 4.9) for cases, and 28.9 (*SD* = 4.4) for controls.

Participant age at the time of urine sample collection ranged from 49 to 69 years for both case and control groups. The mean age of participants that provided urine samples was 61 years for cases, and 60.9 years for controls. The majority (86.8%) of participants were from New Brunswick (99 cases, 33 controls). The remaining samples included 7.9% from Nova Scotia (3 cases, 9 controls) and 5.3% from Prince Edward Island (2 cases, 3 controls). No participants from Newfoundland and Labrador provided urine samples. Only 32.3% of participants provided information regarding primary water sources, with 21.1% reporting municipal or bottled water, and 11.2% reporting well water. Overall, 16.4% of participants had a family history of prostate cancer, and prostate cancer cases had a higher percentage of family history of prostate cancer (31.6%) compared to healthy controls (11.4%). Among the 85.5% of participants that provided BMI information, the mean BMI was 29.7 (*SD* = 4.1) overall, 29.7 (*SD* = 4.1) for cases, and 29.1 (*SD* = 4.5) for controls. Table 1 details participant characteristics for both toenail and urine samples, including age, province of residence, water source, household income, smoking status, family history of prostate cancer, and BMI.

**Table 1:** Participant characteristics of prostate cancer cases and controls from toenail (*n* = 539) and urine (*n* = 152) sample groups, sourced from the Atlantic PATH cohort study (2009-2015).

**Variable**	**Toenail samples** ***n =*** **539**			**Urine samples** ***n =*** **152**		
	**Overall *n =* 539**	**Controls *n =* 400**	**Case *n =* 139**	**Overall *n =* 152**	**Controls *n =* 114**	**Cases *n =* 38**
Age mean (SD)	61.3 (5.0)	61.2 (4.9)	61.7 (5.1)	60.9 (5.2)	60.9 (5.1)	61.0 (5.4)
48–54 *n* (%)	57 (10.6)	42 (10.5)	15 (10.8)	23 (15.1)	17 (14.9)	6 (15.8)
55–71 *n* (%)	485 (89.4)	358 (89.5)	124 (89.2)	129 (84.9)	97 (85.1)	32 (84.2)
Province *n* (%)						
New Brunswick	267 (49.5)	197 (49.3)	70 (50.4)	132 (86.8)	99 (86.8)	33 (86.8)
Newfoundland and Labrador	123 (22.8)	92 (23.0)	31 (22.3)	12 (7.9)	9 (7.9)	3 (7.9)
Nova Scotia	121 (22.4)	90 (22.5)	31 (22.3)	8 (5.3)	6 (5.3)	2 (5.3)
Prince Edward Island	28 (5.2)	21 (5.3)	7 (5.0)	0 (0.0)	0 (0.0)	0 (0.0)
Water source *n* (%)						
Municipal or bottled water	183 (34.0)	142 (35.5)	41 (29.5)	32 (21.1)	24 (18.4)	8 (21.1)
Well water	90 (16.7)	69 (17.3)	21 (15.1)	17 (11.2)	14 (12.3)	3 (7.9)
Unknown	266 (49.4)	189 (47.3)	77 (55.4)	103 (67.8)	76 (66.7)	27 (71.1)
Household income (CAD) *n* (%)						
<49,999	103 (19.1)	79 (19.8)	24 (17.3)	28 (18.4)	21 (18.4)	7 (18.4)
50,000 – 74,999	134 (24.9)	103 (25.8)	31 (22.3)	38 (25.0)	28 (24.6)	10 (26.3)
>75,000	269 (49.9)	194 (48.5)	75 (54.0)	75 (49.3)	57 (50.0)	18 (47.4)
Unknown	33 (6.1)	24 (6.0)	9 (6.5)	11 (7.2)	8 (7.0)	3 (7.9)
Smoking status *n* (%)						
Never smoked	214 (39.7)	156 (39.0)	58 (41.7)	61 (40.1)	43 (37.7)	18 (47.4)
Former smoker	285 (52.9)	215 (53.8)	70 (50.4)	79 (52.0)	61 (53.5)	18 (47.4)
Current smoker	31 (5.8)	23 (5.8)	8 (5.8)	9 (5.9)	8 (7.0)	1 (2.6)
Unknown	9 (1.7)	6 (1.5)	3 (2.2)	3 (2.0)	2 (1.8)	1 (2.6)
Family history of prostate cancer *n* (%)						
No	469 (87.0)	365 (91.3)	104 (74.8)	127 (83.6)	101 (88.6)	26 (68.4)
Yes	70 (13.0)	35 (8.8)	35 (25.2)	25 (16.4)	13 (11.4)	12 (31.6)
BMI mean (SD)	29.1 (4.5)	28.9 (4.4)	29.6 (4.9)	29.7 (4.1)	29.1 (4.5)	29.3 (4.4)
Low/normal weight	49 (9.1)	38 (9.5)	11 (7.9)	15 (9.9)	13 (11.4)	2 (5.3)
Overweight	179 (33.2)	136 (34.0)	43 (30.9)	64 (42.1)	49 (43.0)	15 (39.5)
Obese	149 (27.6)	105 (26.3)	44 (31.7)	51 (33.6)	36 (31.6)	15 (9.9)
Unknown	162 (30.1)	121 (30.3)	41 (39.5)	22 (14.5)	16 (14.0)	6 (15.8)

### Arsenic Speciation Profiles in Toenails and Urine

**Figure 2:**
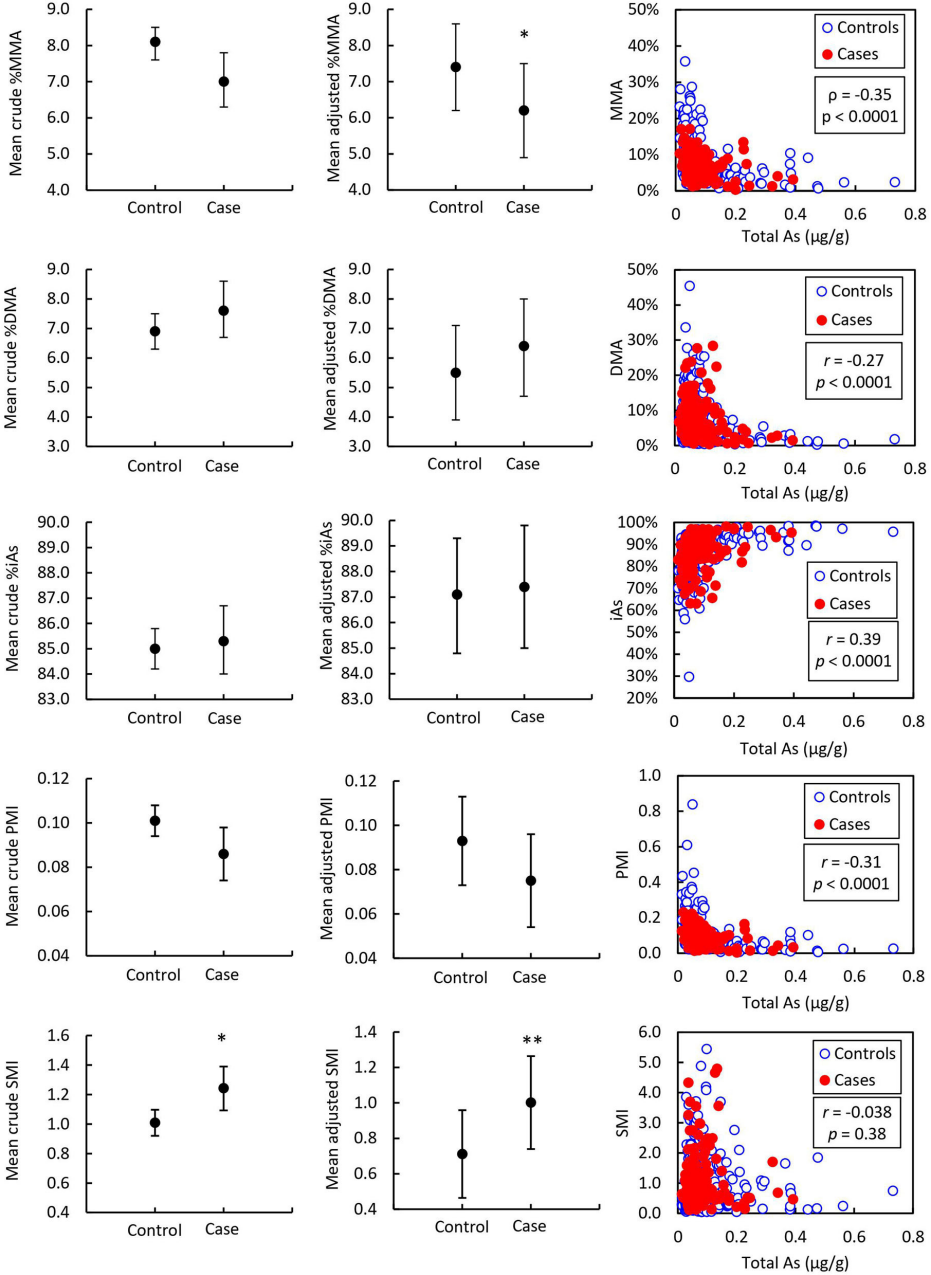
Mean toenail arsenic species in prostate cancer cases (*n* = 139) and controls (*n* = 400) and correlation with total arsenic. Asterisks indicate the statistical significance of the mean difference between prostate cancer cases and healthy controls; * indicates *p* < 0.01. Error bars indicate 95% CI. *r* = Pearson correlation coefficient associated with the correlation between arsenic species and total arsenic. *p* = *p*-value associated with Pearson correlation coefficient.

**Figure 3:**
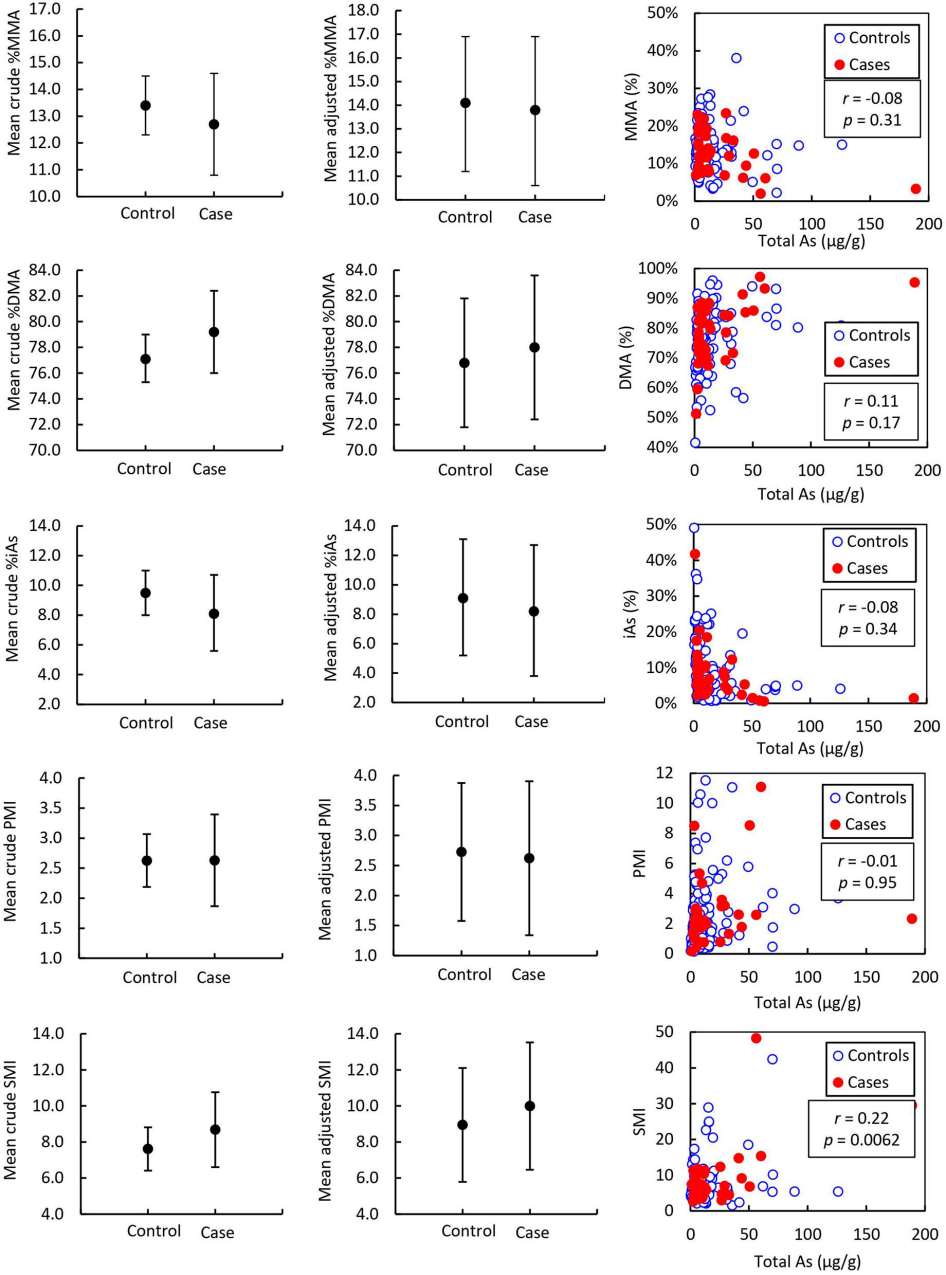
Mean urine arsenic species of prostate cancer cases (*n* = 38) and controls (*n* = 114) and correlation with total arsenic adjusted to urinary creatinine concentration. Error bars indicate 95% CI. *r* = Pearson correlation coefficient associated with the correlation between arsenic species and total arsenic expressed as μg As per g urinary creatinine. *p* = *p*-value associated with Pearson correlation coefficient.

Detailed results from the ANCOVA comparing adjusted mean and standard error (*SE*) As speciation in toenails and urine of prostate cancer cases and healthy controls are reported in Table 2 and illustrated in Figures 2, 3. In toenail samples (Figure 2), the adjusted mean %MMA was 6.2 (95% CI 4.9–7.5) in prostate cancer cases and 7.4 (95% CI 6.2–8.6) in healthy controls, with a corresponding mean difference of −1.18 (*SE* = 0.45) and a statistically significant *p*-value of 0.0087 in the adjusted model after Bonferroni adjustment. There was no significant mean difference in %DMA nor %iAs in toenails between cases and controls. Adjusted mean primary methylation index (PMI, [MMA/iAs]) in toenails was 0.075 (95% CI 0.054–0.096) in prostate cancer cases and 0.093 (95% CI 0.073–0.113) in healthy controls, with a corresponding mean difference of −0.0186 (*SE* = 0.0073) and a *p*-value of 0.011 in the adjusted model. Adjusted mean secondary methylation index (SMI, [DMA/MMA]) in toenails was 1.002 (95% CI 0.739–1.264) in prostate cancer cases and 0.711 (95% CI 0.463–0.959) in healthy controls, with a corresponding mean difference of 0.2906 (*SE* = 0.09) and Bonferroni-adjusted statistically significant *p*-value of 0.0013. In urine samples (Figure 3), there were no significant mean differences in any As speciation measures between prostate cancer cases and healthy controls in neither the crude nor the adjusted models.

**Table 2:** ANOVA (crude) and ANCOVA (adjusted) models of arsenic speciation measures in toenail and urine samples between prostate cancer cases and controls.

**Variable**	**Case mean (SE)**	**Control mean (SE)**	**Crude mean difference (SE)^**a**^**	**Effect size^**b**^**	***p*-value^**c**^**	**Adjusted mean difference (SE)^**d**^**	**Effect size*^***e***^***	***p*-value^**f**^**
Toenails (*n =* 539)								
%MMA	7.0 (3.3)	8.1 (4.7)	−1.03 (0.44)	0.0103	0.018	−1.18 (0.45)	0.0132	0.0087
%DMA	7.6 (5.8)	6.9 (5.7)	0.73 (0.56)	0.0031	0.200	0.82 (0.58)	0.0038	0.1600
%iAs	85.3 (7.6)	85.0 (8.2)	3.0 (0.79)	0.0003	0.700	0.36 (0.82)	0.0004	0.6600
PMI	0.086 (0.046)	0.101 (0.079)	−0.0153 (0.0071)	0.0086	0.031	−0.0186 (0.0073)	0.0123	0.0110
SMI	1.242 (1.054)	1.008 (0.815)	0.234 (0.088)	0.0130	0.008	0.291 (0.090)	0.0197	0.0013
Urine (*n =* 152)								
%MMA	12.7 (5.5)	13.4 (6.0)	−0.66 (1.1)	0.0024	0.55	−0.28 (−1.14)	0.0005	0.80
%DMA	79.2 (9.7)	77.2 (10.1)	2.04 (1.89)	0.0079	0.28	1.18 (2.00)	0.0026	0.55
%iAs	8.1 (7.4)	9.5 (8.1)	−1.38 (1.5)	0.0057	0.36	−0.89 (1.6)	0.0024	0.57
PMI	2.632 (2.292)	2.628 (2.414)	0.0041 (0.4503)	0.0000	0.99	−0.105 (0.461)	0.0004	0.82
SMI	7.609 (5.814)	8.680 (8.206)	1.071 (1.225)	0.0052	0.38	1.044 (1.271)	0.0051	0.41

a *Mean difference between case and matched controls in the crude model, unadjusted for covariates*.

b *Effect size of crude mean difference expressed as partial η^2^*.

c *p-value associated with crude mean difference. Based on the Bonferroni correction method, significance was set at p < 0.01*.

d *Mean difference between case and matched controls in the adjusted model, adjusted for age, province of residence, household income, BMI, smoking status, family history of prostate cancer, and water source*.

e *Effect size of adjusted mean difference expressed as partial η^2^*.

f *p-value associated with adjusted mean difference. Based on the Bonferroni correction method, significance was set at p < 0.01*.

*Missing questionnaire data were treated as unknown*.

ORs and CIs for the binary outcome of prostate cancer history per *SD* change of As speciation measures in toenails and urine samples are detailed in Table 3. In toenail samples, one *SD* increase of %MMA was associated with a 26% less likely chance of having a history of prostate cancer (OR = 0.736, 95% CI 0.584–0.929, *p* = 0.01). One *SD* increase of SMI in toenails was associated with a 38% more likely chance of having a history of prostate cancer (OR = 1.378, 95% CI 1.134–1.675, *p* = 0.001). No ORs calculated from urine samples were significant.

**Table 3:** Association between prostate cancer and arsenic speciation measures in toenail and urine samples.

**Variable**	**Mean (STD)^**a**^**	**Crude odds ratio** ^**b**^ ^,^ ^**c**^	**95% CI**	***p*–value^**d**^**	**Adjusted odds ratio^**c**^^,^^**e**^**	**95% CI**	***p*–value^**f**^**
Toenails (*n =* 539)							
%MMA	7.8 (4.4)	0.767	0.614–0.958	0.019	0.736	0.584–0.929	0.010
%DMA	7.1 (5.7)	1.130	0.939–1.360	0.197	1.155	0.950–1.404	0.148
%iAs	85.1 (8.0)	1.039	0.855–1.264	0.700	1.044	0.853–1.278	0.673
PMI	0.097 (0.072)	0.757	0.588–0.975	0.031	0.730	0.563–0.948	0.018
SMI	1.068 (0.897)	1.272	1.060–1.527	0.010	1.378	1.134–1.675	0.001
Urine (*n =* 152)							
%MMA	13.2 (5.8)	0.889	0.609–1.299	0.544	0.991	0.648–1.515	0.965
%DMA	77.6 (10.0)	1.239	0.842–1.823	0.276	1.144	0.749–1.749	0.533
%iAs	9.1 (8.0)	0.822	0.543–1.245	0.355	0.840	0.532–1.325	0.453
PMI	2.629 (2.376)	1.002	0.693–1.447	0.993	1.013	0.671–1.528	0.952
SMI	7.877 (6.482)	1.162	0.829–1.628	0.384	1.181	0.783–1.782	0.427

a *Mean value and standard deviation of all samples*.

b *Crude regression model, unadjusted for covariates*.

c *Standardized by STD so that OR was calculated per STD change of independent variable*.

d *p-value associated with the crude model. Based on the Bonferroni correction method, significance was set at p < 0.01*.

e *Multiple regression model adjusted for age, province of residence, household income, BMI, smoking status, family history of prostate cancer, and water source. Missing questionnaire data were treated as unknown*.

f *p-value associated with the multiple regression model. Based on the Bonferroni correction method, significance was set at p < 0.01*.

### Metallome Profiles in Toenails and Urine

**Figure 4:**
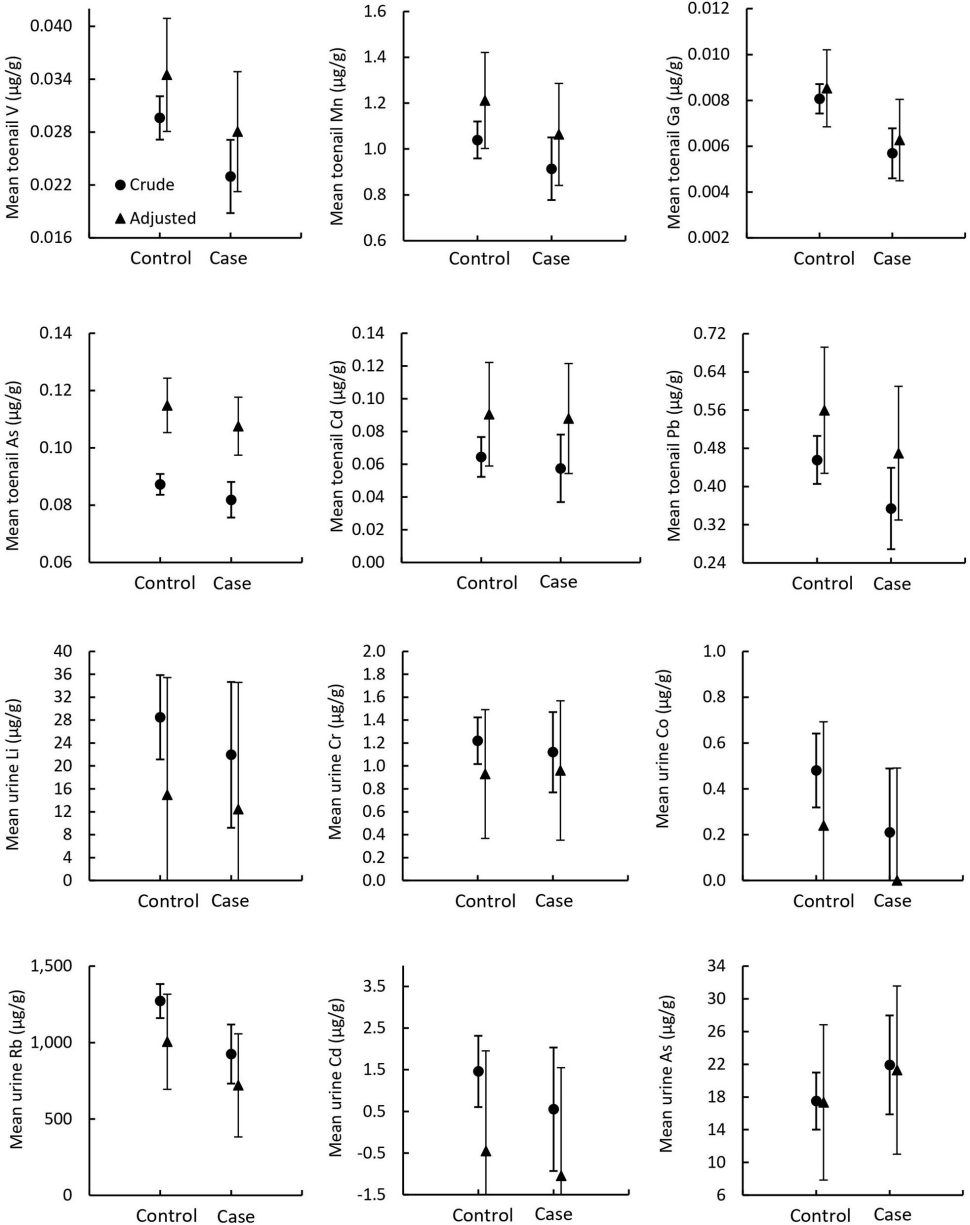
Mean metallome concentrations in toenail and urine samples. Circles indicate crude means from MANOVA models and triangles indicate adjusted means from MANCOVA models. Error bars indicate standard error.

In the toenail sample group, five metals (Li, Ag, Sb, Ba, and Hg) had total concentrations below MDL in most samples and were excluded from statistical analysis. Six metals (V, Mn, Ga, Pb, As, and Cd) were identified for further analysis: V, Mn, Ga, and Pb met the statistical inclusion criteria defined *a priori*, Student's *t*-test *p* < 0.1 (Supplementary Table 3), and As and Cd were included on a theoretical basis as they have been previously associated with prostate cancer (1). In the urine sample group, 12 metals (Mn, Fe, Ni, Cu, Ga, Ag, Sb, Ba, Hg, Pb, Th, and U) had total concentrations below MDL in most samples and were excluded from statistical analysis. A total of six metals were identified for profile analysis in urine samples: Li, Cr, Co, and Rb met the statistical inclusion criteria (Student's *t*-test or Wilcoxon test *p* < 0.1), and As and Cd were included due to their reported association with prostate cancer (1). Results from the MANOVA of metallome profiles comparing mean differences of total metal concentrations in toenails and urine between prostate cancer cases and controls are reported in Table 4 and illustrated in Figure 4. There were no significant differences in mean total metal concentrations between cases and controls for any of the metals tested. The metallomic profiles in toenails samples were not statistically significant in the crude (*p* = 0.63) or adjusted (*p* = 0.72) models. Similarly, in urine samples, the crude and adjusted models ORs and CIs were non-significant and Wilks' lambda *p*-values were 0.13 and 0.17, respectively.

**Table 4:** MANOVA and MANCOVA models of mean difference of total metal concentrations between prostate cancer cases and controls from toenail (*N* = 539) and urine (*N* = 152) sample groups.

**Metal**	**Case mean (SE)**	**Control mean (SE)**	**Crude Mean Difference (SE)^**a**^**	***p*–value^**b**^**	**Adjusted Mean Difference (SE)^**c**^**	***p*–value^**d**^**	**Effect size^**e**^**
Toenails (*n =* 539)				0.63		0.71	
V (μg/g)	0.023 (0.035)	0.030 (0.053)	−0.0066 (0.0048)	0.17	−0.0054 (0.0049)	0.17	0.0037
Mn (μg/g)	0.914 (1.754)	1.039 (1.557)	−0.1252 (0.1589)	0.43	−0.1314 (0.1605)	0.42	0.0012
Ga (μg/g)	0.006 (0.007)	0.008 (0.014)	−0.0024 (0.0013)	0.059	−0.0023 (0.0013)	0.059	0.0069
As (μg/g)	0.082 (0.062)	0.087 (0.077)	−0.0054 (0.0072)	0.45	−0.0079 (0.0074)	0.45	0.0011
Cd (μg/g)	0.057 (0.181)	0.064 (0.262)	−0.0070 (0.0240)	0.77	−0.0024 (0.0245)	0.77	0.0002
Pb (μg/g)	0.354 (0.579)	0.456 (1.116)	−0.1017 (0.0992)	0.30	−0.0948 (0.1023)	0.30	0.0020
Urine (*n =* 152)				0.13		0.14	
Li (μg/g)^f^	21.93 (12.76)	28.49 (90.05)	−6.5548 (14.807)	0.66	−2.474 (15.988)	0.66	0.0014
Cr (μg/g)	1.123 (0.891)	1.217 (2.442)	−0.0939 (0.4069)	0.82	0.0640 (0.4355)	0.82	0.0004
Co (μg/g)	0.208 (0.420)	0.479 (1.965)	−0.2710 (0.3245)	0.40	−0.2358 (0.3549)	0.42	0.0048
As (μg/g)	21.92 (46.15)	17.50 (33.88)	4.4149 (7.0405)	0.53	3.9650 (7.4421)	0.53	0.0029
Rb (μg/g)	924.9 (581.4)	1272.1 (1332.0)	−347.37 (225.02)	0.12	−277.04 (239.58)	0.13	0.0172
Cd (μg/g)	0.547 (2.046)	1.458 (10.456)	−0.9117 (1.7245)	0.59	−0.5794 (1.8795)	0.61	0.0020

a *Mean difference between prostate cancer cases and matched healthy controls from the unadjusted MANOVA model. Wilks' lambda p-value corresponding to the crude toenail and urine models are 0.627 and 0.1297, respectively*.

b *p-value corresponding to the mean difference from the crude MANOVA model*.

c *Mean difference between prostate cancer cases and matched healthy controls from the adjusted MANCOVA model. The model was adjusted for age, province of residence, household income, smoking status, BMI, family history of prostate cancer, and water source. Missing questionnaire data were treated as unknown. Wilks' lambda p-value corresponding to the adjusted toenail and urine models are 0.7193 and 0.172, respectively*.

d *p-value corresponding to the mean difference from the adjusted MANCOVA model*.

e *Effect size expressed as partial η^2^*.

f *Urine metal concentrations expressed as μg per g of urinary creatinine*.

Odds ratios and CIs for the binary outcome of prostate cancer history per *SD* change of selected metal concentrations in toenail and urine samples are detailed in Table 5. No ORs in toenails nor urine samples were significant.

**Table 5:** Association between prostate cancer and selected metal concentrations in toenail and urine samples.

**Variable**	**Mean (STD)^**a**^**	**Crude odds ratio** ^**b**^ ^,^ ^**c**^	**95% CI**	***p*–value^**d**^**	**Adjusted odds ratio** ^**c**^ ^,^ ^**d**^	**95% CI**	***p*–value*^***f***^***
Toenails (*n =* 539)							
V (μg/g)	0.028 (0.049)	0.824	0.621–1.094	0.181	0.848	0.638–1.128	0.257
Mn (μg/g)	1.007 (1.609)	0.917	0.739–1.138	0.432	0.910	0.729–1.137	0.408
Ga (μg/g)	0.007 (0.013)	0.748	0.546–1.025	0.071	0.759	0.553–1.042	0.088
As (μg/g)	0.086 (0.073)	0.923	0.749–1.139	0.456	0.891	0.718–1.106	0.295
Cd (μg/g)	0.063 (0.243)	0.968	0.779–1.203	0.772	0.989	0.795–1.230	0.923
Pb (μg/g)	0.429 (1.006)	0.877	0.678–1.133	0.314	0.881	0.680–1.149	0.357
Urine (*n =* 152)							
Li (μg/g) *^*g*^*	26.85 (78.21)	0.882	0.492–1.582	0.675	0.951	0.576–1.569	0.844
Cr (μg/g)	1.194 (2.159)	0.954	0.639–1.424	0.816	1.027	0.671–1.573	0.901
Co (μg/g)	0.411 (1.717)	0.411	0.069–2.429	0.326	0.273	0.039–1.900	0.190
Rb (μg/g)	1185 (1197)	0.503	0.209–1.208	0.124	0.566	0.244–1.314	0.186
As (μg/g)	18.61 (37.21)	1.114	0.796–1.560	0.530	1.067	0.727–1.567	0.740
Cd (μg/g)	1.230 (9.110)	0.818	0.337–1.990	0.658	0.793	0.215–2.925	0.728

a *Mean value and standard deviation of all samples*.

b *Crude regression model, unadjusted for covariates*.

c *Standardized by STD so that OR was calculated per STD change of independent variable*.

d *p-value associated with the crude model. Based on the Bonferroni correction method, significance was set at p < 0.01*.

e *Multiple regression model adjusted for age, province of residence, household income, BMI, smoking status, family history of prostate cancer, and water source. Missing questionnaire data were treated as unknown*.

f *p-value associated with the multiple regression model. Based on the Bonferroni correction method, significance was set at p < 0.01*.

g *Urine metal concentrations expressed as μg per g of urinary creatinine*.

### Correlation of Arsenic Speciation and Total Arsenic

Pearson correlation coefficients (*r*) for As speciation measures (%MMA, %DMA, %iAs, PMI, and SMI) with total As measured by ICP-MS are reported in Table 6 and illustrated in Figure 2. In toenail samples, %MMA, %DMA, and PMI were significantly negatively correlated with total iAs concentration, with Pearson correlation coefficients of −0.36, −0.27, and −0.31 respectively (*p* < 0.0001). The proportion of iAs was significantly positively correlated with total As concentration in toenails (*r* = 0.39, *p* < 0.0001). SMI was not correlated with total As concentration in toenails. By contrast, in urine (Figure 3), SMI was significantly positively correlated with total As concentration (*r* = 0.22, *p* = 0.0062), but no other As speciation measures were correlated with total As concentration. Figures 2, 3 depict the correlation of As speciation with total As concentrations in toenail and urine samples, respectively.

**Table 6:** Correlation of arsenic speciation measures with total arsenic measured in toenail and urine samples.

**Measure**	**Toenails** ***n =*** **539**		**Urine** ***n =*** **152**	
	**Pearson correlation coefficient^**a**^**	***p*–value^**b**^**	**Pearson correlation coefficient^**c**^**	***p*–value^**d**^**
%MMA	−0.35710	<0.0001	−0.08309	0.31
%DMA	−0.27373	<0.0001	0.11081	0.17
%iAs	0.39227	<0.0001	−0.07858	0.34
PMI	−0.31444	<0.0001	−0.00503	0.95
SMI	−0.03765	0.39	0.2213	0.0062

a *Correlation of As speciation measures with total As in toenails*.

b *p-value associated with Pearson correlation coefficient between As species and total As in toenails*.

c *Correlation of As speciation measures with total As in urine*.

d *p-value associated with Pearson correlation coefficient between As species and total As in urine*.

### Power Comparison for Toenail and Urine Samples

Analysis of covariance models of As speciation using equal numbers of toenail and urine samples (*n* = 140) are presented in Table 7. As speciation profiles, particularly %MMA and PMI, in the toenail sample subset approached statistical significance, while no measure approached significance in the urine sample subset. The effect size of the mean difference in %MMA between prostate cancer cases and controls was larger in the toenail sample subset (${\eta^{2}}_{p}$ = 0.0257, *p* =0.059) than in the urine sample subset (${\eta^{2}}_{p}$ = 0.0013, *p* = 0.67). Similarly, the effect size of the mean difference in PMI between prostate cancer cases and controls was larger in the toenail sample subset (${\eta^{2}}_{p}$ = 0.0238, *p* = 0.069) than in the urine sample subset (${\eta^{2}}_{p}$ = 0.0000, *p* = 0.98).

**Table 7:** ANCOVA models using matched toenail (*n* = 140) and urine (*n* = 140) samples to test the relative statistic power of toenail and urine samples to capture altered arsenic methylation in prostate cancer cases.

**Variable**	**Sample type**	**Mean difference (SE)^**a**^**	**Effect size^**b**^**	***p*–value^**c**^**
%MMA	Toenail	−1.81 (0.96)	0.0257	0.059
	Urine	−0.48 (1.14)	0.0013	0.67
%DMA	Toenail	−0.51 (0.90)	0.0023	0.57
	Urine	1.72 (1.97)	0.0056	0.38
%iAs	Toenail	2.32 (1.49)	0.0176	0.12
	Urine	−1.24 (1.58)	0.0045	0.43
PMI	Toenail	−0.026 (0.014)	0.0238	0.069
	Urine	0.0114 (0.4716)	0.0000	0.98
SMI	Toenail	0.0148 (0.1258)	0.0001	0.91
	Urine	0.5807 (1.2545)	0.0016	0.64

a *Mean difference between prostate cancer cases and controls in ANOVA model*.

b *Effect size expressed as partial η^2^*.

c *p-value associated with a mean difference between prostate cancer cases and controls in ANOVA model*.

## Discussion

### Biomarkers of Arsenic Methylation Capacity

To the best of our knowledge, this is the first study to measure As species in toenails and urine among prostate cancer cases and controls. The finding that the proportions of As species in toenails differ significantly between prostate cancer cases and controls, with cases having significantly lower %MMA and higher SMI than healthy controls, provides evidence for their potential to act as a biomarker in this cancer sub-population. The difference in SMI observed in our work is likely attributed to the lower proportion of MMA in toenails, rather than a change in the proportion of toenail DMA. In urine samples, however, we found no significant difference in the proportions of As species between prostate cancer cases and controls. These findings are not consistent with previous observational work in urine samples from other cancer types, which found a statistically significant association between a higher proportion of MMA in urine and skin cancer (33), lung cancer (58), bladder cancer (35, 37, 38, 58), and breast cancer (36). Some of these studies also found that lower urinary SMI was associated with skin cancer (33), bladder cancer (35, 37, 38), and breast cancer (36). Of the few observational studies that investigate the correlation between iAs exposure and As species in urine, the results are inconclusive. Some found that increased As exposure is associated with increased proportions of urinary MMA, decreased proportions of urinary DMA, and decreased urinary SMI (31, 39), while others found no correlation between total As and proportions of MMA or DMA (58) in urine.

The inconsistencies with previous findings in urine indicate that the storage of As species may differ between toenail and urine samples. Previous studies have only found a consistent correlation of total As between toenail and urine in samples from highly exposed populations where median toenail As is ≥1 μg/g (46), over one order of magnitude higher than the median observed in this study (0.06 μg/g for prostate cancer cases and 0.068 μg/g for controls, see Supplementary Table 3). The difference in observed As species profiles in toenails of prostate cancer cases in this study compared to those observed in the urine of other cancer types may indicate that the kinetic reactions that cause urinary iAs excretion or iAs deposition in nails result in different As species profiles in each type of biospecimen.

Urine is the main pathway through which approximately 75% of absorbed As is excreted and represents an exposure window of up to 3–5 days (46). The remaining iAs retained by the body circulates in the blood and a fraction is incorporated into the keratin matrix of nails, representing an iAs body burden for a period of up to 18 months (46). The present study found no correlation between total As and proportions of iAs, MMA, or DMA in urine, which suggests that urine samples may not capture evidence of altered methylation capacity as a result of iAs exposure in the Atlantic PATH cohort (Table 4). The lack of correlation between total As and As speciation profiles in urine could be explained by the presence of organic As species, such as arsenobetaine (AsB), arsenosugars, and arsenolipids associated with seafood consumption, which may confound the association between total As and As speciation (59). Toenails contain smaller or sometimes undetectable proportions of AsB (60, 61) and there is evidence that AsB and other organoarsenicals associated with seafood are efficiently excreted in urine and do not accumulate in the body (62). This suggests that total As in toenails only reflects exposure to iAs (46) which could make them a more viable biospecimen to use in ascertaining the effect of iAs exposure on iAs methylation capacity. Our results indicate that toenail As speciation profiles are significantly correlated with total As concentrations in toenail samples. Specifically, concentrations of total As in toenails are correlated with increased %iAs, decreased %MMA, decreased %DMA, and by extension, decreased PMI (Table 4). These observations suggest that toenail As species as a biomarker have potential utility for cancer risk assessment associated with iAs exposure.

No epidemiologic studies to date have directly investigated the association between iAs methylation capacity and prostate cancer in humans, but recent studies have found that chronic exposure to iAs is associated with reduced iAs methylation capacity and polymorphisms of genes involved in the methylation of iAs (63, 64). These gene polymorphisms are also associated with an increased risk of cancer (63, 64). *In vitro* studies have found that chronic iAs exposure leads to oxidative DNA damage (65) and decreased capacity to methylate iAs (66, 67), as well as DNA hypomethylation and the transformation of prostate epithelial cells to malignant tumorigenic cells (68–70). The finding in this study that prostate cancer cases had lower toenail %MMA and higher SMI (likely as a result of lower %MMA) than controls supports the hypothesis that altered iAs methylation may be the key to understanding mechanisms of As-induced carcinogenesis. This is consistent with previous results showing that low methylation capacity, as a result of increased iAs exposure, may increase lung cancer occurrence (71), and several observational studies that found altered iAs methylation capacity to be associated with a higher risk of As-induced disease (33–35, 39).

Our findings demonstrate that As speciation profiles of prostate cancer cases significantly differ between toenail and urine samples and that toenails may be well-suited to detect significant differences in %MMA and methylation indices between cases and controls compared to urine. Considering the advantages as a marker of longer-term exposure to iAs, non-invasive collection methods, storage at room temperature for long periods of time, and the affinity of iAs to keratin (48), our findings indicate that toenails are a suitable biospecimen for detecting biomarkers of altered iAs methylation capacity and As speciation profiles that are associated with a history of prostate cancer. These findings warrant further investigation of the association between chronic exposure to iAs, iAs methylation capacity, and carcinogenesis using toenail samples.

### Metallome Profiles

As stated, exposure to environmental metals has been associated with several cancers. Metallomics is an emerging scientific field that draws on the comprehensive analysis of the metallome within a biological specimen. A metallomics approach has not been employed in toenail biomonitoring studies on the identification of specific metallome of the prostate cancer group as a function of environmental metal exposure and the elucidation of their biological functions in cancer pathogenesis. This research determined the complete metallome of toenails and urine. We found no significant differences in mean metal concentrations in toenails or urine between prostate cancer cases and controls. Some observational studies of metals in toenails, however, have found higher total Cd concentrations in the nails of prostate cancer cases compared to healthy controls (72, 73), and that total Mn concentrations were higher in nails from prostate cancer cases compared to healthy controls (73, 74). Results from observational studies measuring trace metals in urine have been inconsistent. Higher urinary total concentrations of Zn, As, sodium (Na), Mg, calcium (Ca), sulfur (S), and strontium (Sr) have been found among prostate cancer cases compared to healthy controls (71, 75). In contrast, Martynko et al. (76) did not find any association between urinary Zn and prostate cancer, as well as for aluminum (Al), boron (B), Cd, Fe, potassium (K), Mn, phosphorus (P), Pb, and silicon (Si). Furthermore, epidemiological studies have indicated that As and Cd exposure are linked with prostate cancer (1), but this study found no significant differences in total As or Cd concentrations in toenail clippings or urine samples from prostate cancer cases relative to controls. The case definition in the present study included those with a history of prostate cancer and does not account for the time of survivorship, which may not account for changes in As body burden, although Cd has a long biological half-life. Future studies will include incident prostate cancer cases, which may clarify the relationship between metallomic profiles in nails or urine and prostate cancer.

### Strengths and Limitations

The strengths of this study lie in its careful selection of appropriately matched cases and controls from the Atlantic PATH cohort study, robust method validation, and the measurement of 23 metals in toenails, 11 metals in urine, and As species in both toenails and urine in a large sample set. In addition, the metallome and As speciation data for the participants of this study will be integrated into the Atlantic PATH database, making these data available for future work involving these participants. This study is cross-sectional in nature as it reflects the baseline period of data collection, but the longitudinal nature of Atlantic PATH allows us to expand upon this work by including incident cases and trends over time in our future work. This is an important aspect of the study, as future research will look to better understand how chronic iAs exposure may influence As speciation patterns in toenails and urine over time and its potential association with the development of various cancers, among other chronic diseases.

The volunteer nature of the cohort means that these findings may not be wholly generalizable outside of Atlantic Canada. There are some notable differences between the Atlantic PATH participants and the general population, including higher socioeconomic status and greater proportions of women, people born outside Canada, and people with university degrees. Participants with higher incomes may be more likely to engage in well water monitoring and remediation. The collection methods associated with toenail and urine biospecimens and the geographic scale of four provinces likely influenced the number of available biospecimens (e.g., the greater number of toenails than urine). Given that iAs methylation efficiency may be related to total iAs exposure, this may have skewed our findings toward the null. Additionally, the questionnaire data used in this study to determine the history of prostate cancer, family history of cancer, smoking status, and household income were self-reported and are subject to recall bias and potentially social-desirability bias. We also acknowledge that whether a person currently has prostate cancer and is undergoing treatment or has since recovered may affect iAs methylation or change As species and metal deposition in toenails.

As stated, human exposure to iAs can occur through contaminated drinking water and consumption of food sources such as rice, other cereals, shellfish, and seaweed. The focus of this study is on the main source of arsenic exposure, which is contaminated drinking water. However, the future questionnaires on diet may be able to better account for iAs consumption regarding rice and fish and clarify the water use habits (e.g., in agriculture, in food production, and for cooking and preparing food at the household level) for estimation of the chronic exposure measured as daily intake (i.e., μg/kg body weight per day) among this population.

### Conclusions and Future Directions

We report novel findings that prostate cancer cases have significantly lower %MMA and higher SMI in toenail samples than healthy controls, and that toenail total As is correlated with altered toenail As speciation profiles. Our data suggest that toenails are a statistically powerful and useful biomarker for the investigation of the health impacts of chronic exposure to iAs. Toenail clippings are viable biomarkers for altered iAs methylation capacity or As species storage, capture a longer exposure window than urine, and are more economical to collect and store long term. The proportion of MMA and SMI in toenails is associated with the history of prostate cancer, although there may be differences by cancer type. This work provides further evidence to support the role of iAs methylation as a mediating mechanism between iAs exposure and carcinogenesis. We found no evidence that total metal concentrations measured in toenail clippings and urine, including As and Cd, were significantly associated with the history of prostate cancer. We plan to clarify metallome associations by using incident prostate cancer cases in future work. Future research will investigate how the metallome and As speciation profiles differ between toenails and urine to better understand iAs toxicokinetics, as well as the strengths and limitations of each biomarker. In addition, previous research has suggested that some metals may provide protective effects against iAs exposure, thus future models will build upon this work by analyzing the inter-relationship between As speciation, total metal concentrations, and cancer. Finally, as these data are cross-sectional, causal relationships cannot be inferred and evidence of an association between iAs exposure and cancer will be strengthened through future work which includes the analysis of incident cancer cases. By identifying how potential risk factors and preventative factors interact, not only with iAs exposure, but with individuals' As metabolism and body burden, this research has the potential to significantly advance our understanding of As carcinogenicity, and thus allow us to help develop and inform targeted population-level strategies to mitigate As-induced disease.

## Data Availability Statement

The original contributions presented in the study are included in the article/Supplementary Material, further inquiries can be directed to the corresponding author. Data access inquiries can be made by contacting info@atlanticpath.ca.

## Ethics Statement

This study was granted ethics approval from Dalhousie University Health Sciences Research Ethics Board (REB #2017-4396) and the University of New Brunswick (REB #014-2018). The participants provided their written informed consent to participate in this study.

## Author Contributions

EK conducted the analytical sample analysis and drafted the manuscript. KH contributed to drafting the manuscript. VB performed the urinary creatinine analysis. YC performed the statistical analysis. ES, GI, AA, TD, and JK contributed to the study design and manuscript revision. JK conceived the original research idea and finalized the manuscript. All authors discussed the results and contributed to the final manuscript.

## Funding

This research was conducted using Atlantic PATH data and biosamples, under application 2018-102 with funding from the Canadian Cancer Society (CCS grant #705566), the New Brunswick Health Research Foundation (NBHRF), Nova Scotia Health Authority (NSHA), and Beatrice Hunter Cancer Research Institute (BHCRI). The data used in this research were made available by the Atlantic PATH study, which is the Atlantic Canada regional component of the CanPath funded by the Canadian Partnership Against Cancer and Health Canada.

## Author Disclaimer

The views expressed herein represent the views of the authors and do not necessarily represent the views of Health Canada.

## Conflict of Interest

The authors declare that the research was conducted in the absence of any commercial or financial relationships that could be construed as a potential conflict of interest.

## Publisher's Note

All claims expressed in this article are solely those of the authors and do not necessarily represent those of their affiliated organizations, or those of the publisher, the editors and the reviewers. Any product that may be evaluated in this article, or claim that may be made by its manufacturer, is not guaranteed or endorsed by the publisher.

## Abbreviations

ANCOVA: Analysis of covariance
ANOVA: Analysis of variance
DMA: Dimethylarsinic acid
HPLC: High-performance liquid chromatograph
iAs: inorganic arsenic
ICP-MS: Inductively coupled plasma-mass spectrometer
MANCOVA: Multivariate analysis of covariance
MANOVA: Multivariate analysis of variance
MMA: Monomethylarsonic acid
PMI: Primary methylation index
SMI: Secondary methylation index.
