# Supplementary material for: Arsenic Speciation and Metallomics Profiling of Human Toenails as a Biomarker to Assess Prostate Cancer Cases: Atlantic PATH Cohort Study
Source: Front Public Health. 2022 Jul 7;10:818069. doi: 10.3389/fpubh.2022.818069 (PMC9301242; doi:10.3389/fpubh.2022.818069)
Supplement: Supplementary file 1 [file Data_Sheet_1.pdf]

Supplementary Material

1 Analytical Methods

1.1 Sample preparation

All reagents used were trace metal grade or better quality. All water used, including that used to prepare aqueous solutions, was deionized with 18.2 MΩ-cm resistivity, dispensed from a Milli-Q Advantage A10 unit (MilliporeSigma, ON Canada). Concentrated nitric acid (HNO3, Fisher Scientific, NJ USA) and 30% (v/v) hydrogen peroxide (H2O2, Sigma-Aldrich, MO USA) were used to dissolve toenail clipping samples during microwave digestion. A multi-element tuning solution (P/N THERMO-4AREV-500M, Inorganic Ventures, VA USA) was used to perform ICP-MS tuning. Arsenite (As3+, Sigma-Aldrich, MO USA), arsenate (As5+, Sigma-Aldrich, MO USA), monomethylarsenic acid (Chem Service, PA USA), and dimethylarsenic acid (Chem Service, PA USA) were used to prepare calibration standards for the HPLC-ICP-MS analysis of arsenic speciation, and arsenobetaine (AsB, Sigma Aldrich, MO USA) was used to prepare the internal standard for speciation analysis. All calibration and internal standards were prepared in 1% (v/v) nitric acid to match the concentration in toenail samples. Ammonium carbonate ([NH4]2CO3, 99.999%, Fisher Scientific, ON Canada) was used to prepare mobile phases for the HPLC-ICP-MS analysis.

Toenail clipping samples were weighed using an analytical balance (model Pinnacle-114, Denver Instruments, CO USA) to determine a "wet" weight (i.e., sample mass prior to cleaning and drying) of approximately 50 mg. Where available sample mass was less than 50 mg, the entire sample was used. After weighing, toenail clipping samples were transferred to 10 mL quartz digestion vessels (CEM Corporation, NC USA). Samples were cleaned by sonicating in acetone (99.5%, Acros Organics, NJ USA) for 5 min, rinsing with acetone, then sonicating in water for 5 min. Samples were subsequently rinsed with water three times before being placed in a Heratherm 60 L gravity convection oven (Thermo Scientific, MA USA) at 105°C overnight. Once dry, samples were reweighed to determine dry mass. The average dry mass was 31.9 mg. Toenail clipping samples were completely dissolved using a Discover SP-D microwave digestor (CEM Corporation, NC USA) in a solution of 100 µL concentrated nitric acid (Fisher Scientific, NJ USA), 400 µL water, and 500 µL hydrogen peroxide (Sigma Aldrich, MO USA). Each digestion set included three method blanks. The microwave digestion method was 300 W power, 5 min ramp time to 165°C, and held for 4 min. After digestion, samples were diluted to 10 mL with DI water for a final nitric acid concentration of 1% (v/v), then transferred to 15 mL polypropylene tubes (FroggaBio, ON Canada). For speciation analysis, a 995 µL aliquot of each sample was transferred to a 1.8 mL polypropylene vial (Thermo Scientific, MA USA), and 5 µL of 20 µg/L AsB (Sigma Aldrich, MO USA) in 1% v/v nitric acid was added as an internal standard. Samples were vortexed (model Maxi Mix I, Thermo Scientific, MA USA) immediately before HPLC-ICP-MS analysis to ensure homogeneity.

Urine samples were first vortexed to ensure homogeneity, then a 0.5 mL aliquot was diluted 5x in 1% (v/v) nitric acid to a volume of 2.5 mL. Urine samples were filtered through a 0.45 µm cellulose acetate filter (Sarstedt, NC USA) before being added to a 15 mL polypropylene tube (FroggaBio, ON Canada) and diluted with 1% (v/v) nitric acid to a final volume of 5.5 mL and a dilution factor of 11. For speciation analysis, a 1 mL aliquot of each diluted sample was transferred to a 1.8 mL polypropylene

vial (Fisher Scientific, ON Canada). Samples were vortexed immediately before analysis to ensure homogeneity.

1.2 Creatinine Measurement

Creatinine concentration in urine was measured using Jaffe's method. An eight-point calibration curve was prepared using a serial dilution of creatinine standard (Sigma-Aldrich, ON Canada) in DI water between 0.31 mg/dL and 20 mg/dL. Urine samples were diluted 40 times. Picric acid solution was prepared by adding 65 µL of picric acid (Sigma-Aldrich, ON Canada) to 50 mL of DI water. Sodium hydroxide (NaOH) solution was prepared by adding 400 mg of NaOH (Fisher Scientific, ON Canada) to 10 mL of DI water. Alkaline picric acid solution was then prepared by adding 7.5 mL of NaOH solution to 37.5 mL of picric acid solution. A 50 µL volume of standards, urine samples, and method blanks was added to 100 µL of alkaline picric acid solution in 96-well plates (VWR, ON Canada), then incubated at room temperature for 30 min. Absorbance was measured at 490 nm using a microplate reader (Synergy H1, BioTek, VT USA).

1.3 Metallome Analysis

The concentrations of 23 metals (Li, V, Cr, Mn, Fe, Co, Ni, Cu, Zn, Ga, As, Se, Rb, Sr, Ag, Cd, Sb, Ba, Hg, Tl, Pb, Th, and U) in toenail clipping and urine samples were measured using an inductively coupled plasma-mass spectrometer (ICP-MS, iCAP Q, Thermo Scientific, MA USA) outfitted with a quartz torch, PFA nebulizer, and quartz spray chamber. The torch position, nebulizer flow rate, and lens voltages were tuned daily according to the instructions of the manufacturer using a multi-element tuning solution (P/N THERMO-4AREV-500M, Inorganic Ventures, VA USA). The sample introduction system consisted of an ESI SC-4 DX autosampler and FAST valve (Elemental Scientific, NE USA) to facilitate on-line addition of a 50 µg/L Sc internal standard (AccuStandard, CT USA) in 1% (v/v) nitric acid. Measurements were performed in kinetic energy discrimination (KED) mode using high purity He (>99.999%) as the collision gas with the exception of Se, which was measured in standard (STD) mode. A multi-element calibration standard (P/N IV-ICPMS-71A, Inorganic Ventures, VA USA) was diluted to 0.01, 0.05, 0.1, 0.5, 1, 5, 10, 50, and 100 µg/L in 1% (v/v) nitric acid to form a 9-point calibration curve. Quality control check standards (1 and 10 µg/L) were measured every 15 samples. Qtegra Intelligent Scientific Data Solution software (version 2.7, Thermo Scientific, MA USA) was used to collect and process data from ICP-MS analysis.

1.4 Arsenic Speciation Analysis

Arsenic speciation analysis was completed using a high-performance liquid chromatograph (HPLC, SpectraSYSTEM, Thermo Scientific, MA USA), outfitted with a P4000 pump, AS3000 autosampler, and SN4000 interface module, paired directly to an ICP-MS. The injection of each sample solution and subsequent measurement was coordinated using the Qtegra Intelligent Scientific Data Solution software (version 2.7, Thermo Scientific, MA USA). An anion exchange column (IonPac AS7, 250x2 mm, Thermo Scientific, MA USA) and guard column (IonPac AG7, 50x2 mm, Thermo Scientific, MA USA) were used to separate arsenic species in toenail clipping and urine samples. Ammonium carbonate was used as the mobile phase using a gradient solution between 20 mM and 200 mM to achieve good separation for all arsenic species measured. A calibration stock solution was made by mixing individual As^{3+} (Sigma-Aldrich, MO USA), As^{5+} (Sigma-Aldrich, MO USA), MMA (Chem Service, PA USA), and DMA (Chem Service, PA USA) standards. The stock solution was then diluted to 0.02, 0.05, 0.1, 0.2, 0.5, 1, and 2 µg/L in 1% (v/v) nitric acid to form a 7-point calibration curve. An

AsB standard (Sigma Aldrich, MO USA) was diluted to 20 µg/L in 1% (v/v) nitric acid and 5 µL was added to each sample as an internal standard. The use of hydrogen peroxide for toenail digestion was assumed to have converted As3+ to As5+, as no As3+ was detected in toenail samples, which was confirmed using certified reference materials (CRMs).

1.5 Method Validation

The As speciation analytical method was established and validated prior to the present analyses (Smith et al., 2022). Two certified reference materials (CRMs) were employed in order to validate the results of metal concentration measurement by ICP-MS and arsenic speciation analysis by HPLC-ICP-MS: NIES No. 13 Human Hair (National Institute for Environmental Studies, Tsukuba, Japan) and NIST 2669 Arsenic Species in Frozen Urine (National Institute of Standards and Technology, ML USA), respectively. There is currently no certified reference material for metals nor arsenic speciation in toenails. As such, these CRMS were deemed the best alternatives.

NIES No. 13: Human Hair samples were prepared by weighing approximately 50 mg of the homogenized hair powder and transferring to a 10 mL quartz digestion vessel (CEM Corporation, NC USA). Samples were then dried, weighed, digested, and diluted using the same protocol as toenail clipping samples. Results are reported in Supplemental Materials Table A1.

NIST 2669: Arsenic Species in Frozen Urine exists in two concentration levels (Davis et al. 2010). At each level, samples were prepared by thawing a vial of each level at room temperature, then gently inverting each vial several times to ensure homogeneity. 200 µL of each level was added to a 10 mL quartz digestion vessel and digested using the same protocol as the toenail clipping samples. After digestion, the samples were diluted to 10 mL with DI water. As urine contains higher concentrations of MMA and DMA than nails, the resulting dilution factor of 20 times was purposely chosen to result in MMA and DMA concentrations similar to those observed in toenail clipping samples. Results are reported in Supplemental Materials Table A1. Results of As speciation analysis of NIST 2669 following digestion with nitric acid and hydrogen peroxide confirmed that As3+ species are converted to As5+ during digestion.

The method detection limits (MDLs) were calculated following United States Environmental Protection Agency (EPA) procedures (EPA 2016). Seven method blanks of 1% v/v nitric acid carried through the entire sample preparation procedure alongside toenail samples were used to calculate the MDL for metals and iAs species. The blanks did not contain MMA nor DMA, so seven replicates of the lowest level calibration standard (0.02 µg/L in 1% v/v nitric acid) were used to calculate the MDL for MMA and DMA. MDLs are reported in Supplemental Materials Table A2. 43 samples were below the MDL for MMA, 10 samples were below the MDL for iAs, and 80 samples were below the MDL for DMA, which corresponds to 7.8%, 1.8%, and 14.4% of the 576 total samples, respectively. As species measurements that fell below MDL were replaced with the $MDL/\sqrt{2}$ for that species before being normalized by sample mass. Previous studies reported that this method does not introduce bias when the percentage of values below MDL is below 25% (Barescut et al. 2011; Croughan and Egeghy 2003; Hites 2019; Verbovšek 2011).

Toenail clipping samples whose mass was less than 5 mg were most likely to have As species concentrations below MDL. Among the 19 samples whose mass was less than 5 mg, 14 had MMA concentration below MDL, 10 had iAs concentration below MDL, and 14 had DMA concentration below MDL, corresponding to 73.7%, 52.6%, and 73.5% of the 19 samples, respectively. Comparison of total As measurement by ICP-MS and total As species measurement by HPLC-ICP-MS showed that

replacement of values below MDL with MDL/√2 resulted in overestimation of total arsenic for samples with mass less than 5 mg. As a result, samples whose mass was less than 5 mg were excluded from statistical analysis. Of the 19 excluded samples, 3 were prostate cancer cases and 16 were healthy controls.

1.6 Urinary Analysis Normalization Strategies

Urine concentrations may vary due to individual differences in water intake and other physiological factors. Normalizing the measurements of metals and arsenic species in urine is therefore necessary to compare measurements between participant groups. Numerous normalization strategies have been proposed, including by urine volume, osmolality, specific gravity, creatinine concentration, and total useful signal. A study by Warrack et al. (2009) comparing normalization by urine volume, osmolality, creatinine concentration, and total useful signal found that variability of analyses was highly dependent on normalization method

Urinary creatinine output is relatively constant under normal conditions, and it has become common practise to normalize urinary analyses with this method. For this reason, this strategy was utilized in this study. However, there are some conditions under which normalization to creatinine would be inappropriate, such as cases where kidney impairment may cause deviation from typical creatinine output.

1.7 Statistical Analysis

Differences in study participant characteristics between prostate cancer cases and controls were compared with mean and standard deviation (SD) for continuous variables, and frequency and percentage for categorical variables. To compare profiles of As speciation and metallomes between cases and controls, ANCOVA and MANOVA models were chosen. In the case of As speciation profiles, ANCOVA models were chosen as the variables may be interrelated; to account for the inflated type I error, a Bonferoni adjustment was made to the significance level (α=0.05/(number of comparisons)= 0.01). For total metallomes profile analysis, MANOVA was selected as the variables are not significantly correlated and it reduces the likelihood of type I error from multiple tests. Model significance was evaluated using Wilks' lambda criterion, which is presented alongside estimated mean difference with accompanying p-values and effect size expressed as partial η2 (ηp2; Fritz et al. 2012). Individual metal variables were selected for MANOVA using the Student's t-test or the Wilcoxon test defined a priori, with model inclusion criteria being either a reported association with prostate cancer or $p < 0.1$. In both ANCOVA and MANOVA models, mean differences in i) iAs species and methylation capacity measures and ii) total metallomes were compared between prostate cancer cases and controls with crude and adjusted analyses. In the adjusted model, covariates were selected if they were associated with the outcome or prostate cancer, and included age, province, BMI, smoking status, family history of prostate cancer, and water source. To determine whether total As was linked with methylation, Pearson correlation coefficients were calculated. These analyses were performed independently in our urine and toenail datasets. A separate, secondary analysis was performed to compare the relative statistic power of measuring iAs speciation in toenails and urine. This analysis replicated the previously described ANOVA models amongst a subset of the participants who provided both urine and toenail samples (n=140). Data management and analyses were performed with SAS statistical package version 9.4 (SAS Institute, NC USA). Unless otherwise specified, all tests were two-sided and evaluated with α=0.05. Questionnaire data were categorized, and missing observations were labeled unknown in the adjusted ANCOVA and MANCOVA models.

1.8 References

Barescut J., Lariviere D., Stocki T., Wood M.D., Beresford N.A., Copplestone D. (2011). Limit of detection values in data analysis: do they matter? Radioprotection, 46(6): S85-S90. doi: 10.1051/radiopro/20116728s.

Croughan C.W., Egeghy P.P. 2003. Methods of dealing with values below the limit of detection using SAS. Presented at Southeastern SAS User Group, St. Petersburg, FL, September 22-24, 2003.

Davis W.C., Zeisler R., Sieber J.R., Yu L.L. (2010). Methods for the Separation and Quantification of Arsenic Species in SRM 2669: Arsenic Species in Frozen Human Urine. Anal Bioanal Chem, 396: 3041-3050. doi: 10.1007/s00216-010-3541-y. PMID: 20186533.

Fritz C.O., Morris P.E., Richler J.J. (2012). Effect size estimates: current use, calculations, and interpretation. J Exp Psychol Gen, 141(1): 2. doi: 10.1037/a0024338. PMID: 21823805.

Hites R.A. (2019). Correcting for Censored Environmental Measurements. Environ Sci Technol, 53: 11059-11060. doi: 10.1021/acs.est.9b05042. PMID: 31509396.

Smith, N.K., Keltie, E., Sweeney, E., Weerasinghe S., MacPherson K., Kim, J.S. (2022). Toenail speciation biomarkers in arsenic-related disease: a feasibility study for investigating the association between arsenic exposure and chronic disease. Ecotoxicol. Environ. Saf. 232: 113269. doi: 10.1016/j.ecoenv.2022.113269. PMID: 35144129.

United States Environmental Protection Agency (EPA). (2016). Definition and Procedure for the Determination of the Method Detection Limit, Revision 2. epa.gov/sites/production/files/2016-12/documents/mdl-procedure_rev2_12-13-2016.pdf. [Accessed February 25, 2020].

Verbovšek T. (2011). A comparison of parameters below the limit of detection in geochemical analyses by substitution methods. RMZ – M&G, 58: 393-404.

Warrack B.M., Hnatyshyn S., Ott K.H., Reily M.D., Sanders M., Zhang H., et al. (2009). Normalization strategies for metabonomic analysis of urine samples. J Chromatogr B, 877(5-6): 547-552. doi: 10.1016/j.chromb.2009.01.007. PMID: 19185549.

Yoshinaga J., Morita M., Okamoto K. (1997). New human hair certified reference material for methylmercury and trace elements. Frasenius J Anal Chem, 357:279-283. doi: 10.1007/s002160050153.

2 Supplemental Tables

Supplemental Table 1. Measured total metal concentrations in NIES No. 13 and arsenic species measurements in NIST 2669.

CRM	Analyte	Analysis method	Measured concentration (µg/g)	Reference concentration (µg/g)	Certified or reference value[a]
NIES no. 13	V	ICP-MS	0.20 ± 0.02	0.27	Reference
	Mn	ICP-MS	3.55 ± 0.29	3.9	Reference
	Fe	ICP-MS	140 ± 17	140	Reference
	Co	ICP-MS	0.071 ± 0.012	0.07	Reference
	Cu	ICP-MS	15.7 ± 1.4	15.3 ± 1.3	Certified
	Zn	ICP-MS	170 ± 12	172 ± 11	Certified
	As	ICP-MS	0.11 ± 0.009	0.1	Reference
	Se	ICP-MS	1.88 ± 0.3	1.79 ± 0.17	Certified
	Cd	ICP-MS	0.25 ± 0.02	0.23 ± 0.03	Certified
	Pb	ICP-MS	5.2 ± 0.4	4.6 ± 0.4	Certified
NIST 2669 Level I	Total As	ICP-MS	22.26 ± 0.42	22.2 ± 4.8	Reference
	As^{5+}	HPLC-ICP-MS	2.35 ± 0.20	2.41 ± 0.30	Certified
	MMA	HPLC-ICP-MS	1.84 ± 0.19	1.87 ± 0.39	Certified
	DMA	HPLC-ICP-MS	3.46 ± 0.07	3.47 ± 0.41	Certified
NIST 2669 Level II	Total As	ICP-MS	47.96 ± 0.34	50.7 ± 6.3	Reference
	As^{5+}	HPLC-ICP-MS	6.06 ± 0.30	6.16 ± 0.95	Certified
	MMA	HPLC-ICP-MS	6.77 ± 0.29	7.18 ± 0.56	Certified
	DMA	HPLC-ICP-MS	24.9 ± 0.6	25.3 ± 0.7	Certified

[a]"Reference" indicates that the reference concentration is given by the CRM provider for information only and does not meet the criteria for a certified reference value. "Certified" indicates that the reference concentration given by the CRM provider has been independently verified by at least 4 analytical methods and meets the criteria for a certified reference value.

Supplemental Table 2. Method detection limits (MDLs) for total metal and arsenic speciation analysis. Metal and iAs MDLs were calculated according to EPA procedure using method blanks. MMA and DMA MDLs were calculated using 7 replicates of the lowest level calibration standard (0.02 μg/L).

Analyte	MDL (μg/L)	MDL (μg)[a]	MDL (μg/g)[b]
V	0.009	0.00009	0.003
Cr	0.33	0.0033	0.10
Mn	0.081	0.0008	0.025
Fe	5.8	0.058	1.83
Co	0.027	0.0003	0.009
Ni	0.35	0.0035	0.11
Cu	0.79	0.0079	0.25
Zn	3.7	0.037	1.16
Ga	0.0047	0.00005	0.0015
As	0.011	0.00011	0.0035
Se	0.019	0.0002	0.0060
Rb	0.033	0.00033	0.010
Sr	0.10	0.0010	0.032
Cd	0.016	0.00016	0.0051
Tl	0.0003	0.000003	0.0001
Pb	0.15	0.0015	0.047
Th	0.0007	0.000007	0.00022
U	0.0007	0.000007	0.00022
MMA	0.0049	0.000049	0.0015
DMA	0.0045	0.000045	0.0014
iAs	0.017	0.00017	0.0052

[a] MDL in μg calculated by multiplying the MDL in μg/L by the total sample volume. Total sample volume was 0.010 L for metal analysis and 0.01005 L for arsenic speciation analysis.
[b] MDL in μg/g calculated by dividing the MDL in μg by the average sample mass (0.0391 g).

Supplemental Table 3. Preliminary total metal concentrations in toenail and urine data used to determine candidates for statistical analysis.

Metal	Case Mean	Case Median (IQR[a])	Control Mean	Control Median (IQR[a])	T-test *p*-value	Wilcoxon Test *p*-value
Toenails (μg/g, n=539)						
V	0.023 ± 0.035	0.013 (0.018)	0.03 ± 0.053	0.016 (0.024)	0.099	0.061537
Cr	0.439 ± 0.766	0.246 (0.299)	0.534 ± 1.347	0.245 (0.361)	0.3082	0.446699
Mn	0.914 ± 1.754	0.42 (0.563)	1.039 ± 1.557	0.508 (0.894)	0.4565	0.082629
Fe	48.042 ± 117.74	20.126 (35.467)	61.647 ± 224.344	22.455 (30.449)	0.3655	0.15121
Co	0.021 ± 0.028	0.011 (0.017)	0.023 ± 0.03	0.013 (0.02)	0.4454	0.325236
Ni	25.18 ± 69.393	4.076 (17.135)	31.511 ± 78.541	6.154 (17.216)	0.3717	0.200247
Cu	5.644 ± 4.374	4.267 (2.677)	5.556 ± 4.041	4.389 (2.411)	0.8369	0.840496
Zn	118.511 ± 28.636	114.813 (30.463)	117.292 ± 59.258	111.958 (27.275)	0.7503	0.215258
Ga	0.006 ± 0.007	0.004 (0.004)	0.008 ± 0.014	0.004 (0.006)	0.0114	0.074616
As	0.082 ± 0.062	0.06 (0.054)	0.087 ± 0.077	0.068 (0.051)	0.407	0.399579
Se	1.054 ± 0.531	0.986 (0.223)	1.034 ± 0.562	0.952 (0.222)	0.7184	0.185597
Rb	0.608 ± 0.489	0.499 (0.486)	0.643 ± 0.582	0.503 (0.499)	0.4882	0.739851
Sr	1.295 ± 2.601	0.548 (0.908)	1.28 ± 2.747	0.636 (0.952)	0.9555	0.653938
Cd	0.057 ± 0.181	0.013 (0.026)	0.064 ± 0.262	0.014 (0.034)	0.7294	0.458861
Tl	0.001 ± 0.002	0 (0)	0.001 ± 0.001	0 (0)	0.5111	0.75805
Pb	0.354 ± 0.579	0.181 (0.308)	0.456 ± 1.116	0.171 (0.299)	0.1719	0.794847
Th	0.009 ± 0.018	0.004 (0.006)	0.009 ± 0.015	0.003 (0.006)	0.9873	0.699679
U	0.014 ± 0.039	0.003 (0.007)	0.015 ± 0.038	0.003 (0.009)	0.6864	0.294294
Urine (μg/L, n=152)						
Li	21.93 ± 12.76	19.42 ± 17.09	28.49 ± 90.05	14.00 ± 14.00	0.4518	0.057689
V	0.300 ± 0.481	0.211 ± 0.203	0.403 ± 1.266	0.188 ± 0.211	0.4695	0.843422
Cr	1.123 ± 0.891	0.925 ± 1.090	1.217 ± 2.442	0.614 ± 0.852	0.7290	0.108447
Co	0.208 ± 0.420	0.104 ± 0.133	0.479 ± 1.965	0.113 ± 0.253	0.1695	0.216775
Zn	409.7 ± 1005.0	210.8 ± 203.7	767.6 ± 4296.3	199.4 ± 244.4	0.4111	0.147099

As	21.92 ± 46.15	5.99 ± 17.19	17.50 ± 33.88	7.47 ± 12.23	0.5895	0.826842
Se	32.62 ± 24.69	29.31 ± 31.25	39.95 ± 93.52	25.10 ± 25.02	0.4479	0.448726
Rb	924.9 ± 581.4	802.1 ± 447.9	1272.1 ± 1332.0	955.7 ± 614.8	0.0280	0.032734
Sr	79.69 ± 54.93	57.22 ± 83.28	104.7 ± 180.0	73.16 ± 75.89	0.1912	0.501076
Cd	0.547 ± 2.046	0.128 ± 0.181	1.458 ± 10.456	0.162 ± 0.439	0.3795	0.436146
Tl	0.119 ± 0.097	0.082 ± 0.066	0.128 ± 0.113	0.100 ± 0.078	0.6342	0.426231

[a]Interquartile range
